# Fuel loads acquired at a stopover site influence the pace of intercontinental migration in a boreal songbird

**DOI:** 10.1038/s41598-017-03503-4

**Published:** 2017-06-13

**Authors:** Camila Gómez, Nicholas J. Bayly, D. Ryan Norris, Stuart A. Mackenzie, Kenneth V. Rosenberg, Philip D. Taylor, Keith A. Hobson, Carlos Daniel Cadena

**Affiliations:** 1 Laboratorio de Biología Evolutiva de Vertebrados, Departamento de Ciencias Biológicas, Universidad de los Andes, Bogotá, Colombia; 2 SELVA: Investigación para la conservación en el Neotropico, Bogotá, Colombia; 3 Department of Integrative Biology, University of Guelph, Guelph, Ontario Canada; 4 Bird Studies Canada, Port Rowan, Ontario Canada; 5 Cornell Lab of Ornithology, Ithaca, New York USA; 6 Acadia University, Wolfville, Nova Scotia Canada; 7 Environment and Climate Change Canada, Saskatoon, Saskatchewan Canada; 8 Department of Biology, University of Western Ontario, London, Ontario Canada

## Abstract

Long-distance migratory organisms are under strong selection to migrate quickly. Stopovers demand more time than flying and are used by individuals to refuel during migration, but the effect of fuel loads (fat) acquired at stopover sites on the subsequent pace of migration has not been quantified. We studied stopover behaviour of Grey-cheeked Thrush (*Catharus minimus*) at a site in northern Colombia and then tracked their migration using an intercontinental radio-telemetry array. Tracking confirmed long-distance flights of more than 3000 km, highlighting the key importance of a single stopover site to the migration strategy of this species. Our results suggest that these songbirds behave as time-minimizers as predicted by optimal migration theory, and that fuel loads acquired at this South American stopover site, together with departure date, carry-over to influence the pace of migration, contributing to differences in travel time of up to 30 days in birds subsequently detected in the U. S. and Canada. Such variation in the pace of migration arising from a single stopover site, likely has important fitness consequences and suggests that identifying important fuelling sites will be essential to effectively conserve migratory species.

## Introduction

Although migration is an adaptive behaviour in a wide range of animals^1–3^, it is also thought to impose significant costs on individuals^4^. Studies on various migratory birds^5–7^, mammals^8^ and fish^9^ provide evidence that mortality can be higher during migration than during stationary periods of the annual cycle. In addition, work on birds^10, 11^ and insects^12^ indicates that migrating individuals often undergo significant metabolic and behavioural adjustments to fulfil the high energetic demands of migration. Time spent and energy used during migration can also determine subsequent breeding success^10, 12–15^, emphasizing the high costs that individuals pay when migrating. Because migration is costly, migratory organisms are expected to maximize their fitness behaviourally via minimizing either the time spent, energy consumed, or the risks incurred during migratory journeys^16, 17^.

In terms of time, the highest cost of migration is generally thought to be experienced during stopovers rather than during periods of flight^18, 19^, and birds rely on the time spent at stopover sites to rest and refuel for the next leg of their journeys^20^. Optimal migration theory provides a framework to study stopover behaviour and its consequences by testing whether migrants are time- or energy-minimizers using data on fuelling rate, stopover duration, fuel loads and potential flight ranges^17^. Individuals attempting to minimize the overall time spent on migration are expected to maximize the amount of fuel they can acquire at each stopover in the shortest time possible. A key consequence of this strategy is that it maximizes the distance that can be flown between stopovers^18, 21^. Consequently, the fuel loads (amount of fat carried) of a time-minimizer should be tightly linked to local conditions at stopover sites as well as to the conditions expected ahead because these conditions influence fuelling rates^18, 21^. Furthermore, stopover durations in time-minimizers are expected to have been shaped by or to respond directly to experienced fuelling conditions^17, 18^. Larger departure fuel loads should allow for longer flights and a faster overall pace of migration because individuals acquiring sufficient fuel in the shortest time possible will need to make fewer stopovers and be able to take more direct routes to their destination, including being able to fly over physical barriers or large areas of unsuitable habitat such as deserts or oceans rather than circumventing these areas^22^.

There are two types of energy-minimizers: those that minimize the cost of transporting large fuel loads per unit distance, and those that attempt to minimize the total energy spent on migration^18^. The first type (transport energy-minimizers) avoid the costs of carrying excess baggage by storing the minimum amount of fuel required to reach the next closest stopover site^18^. The second type (total energy-minimizers) minimize the total energy cost of migration by minimizing the energy expenditure during stopover as well as the flight costs between their breeding and non-breeding destinations^18^. When possible, both types of energy-minimizers avoid crossing large areas of unsuitable habitat, which would require carrying large fuel loads, and this is expected to make their routes more circuitous compared to those of time-minimizers^22^. It follows that transport energy-minimizers should show no correlation between fuelling rate and fuel loads, and that their migratory journey should include multiple short stops along the way^18, 23^. Thus, unlike time-minimizers, the pace of migration in transport energy-minimizers would not be influenced by fuel loads or refuelling rate^18^. Total energy-minimizers are expected to be influenced by fuelling rates like time-minimizers but they should attain lower fuel loads and make shorter flights compared to time-minimizers^18^. Consequently, the slope of the relationship between fuelling rate and fuel load is expected to be less pronounced in energy-minimizers^18^. Furthermore, at high fuelling rates (above 0.03 LBM/day), this relationship should level off in total energy minimizers and not in time minimizers^18^.

Evidence from refuelling rates at stopover sites suggests long-distance migratory songbirds are either time-minimizers or total-energy-minimizers^17^. For example, six species of Nearctic-Neotropical migrants^24^ as well as four species of Afro-Palearctic migrants^25–27^ showed positive correlations between fuel loads and fuel deposition rates acquired at stopover sites. Also, there are clear benefits of early arrival at both the breeding^14, 28, 29^ and stationary non-breeding grounds^30–32^, suggesting that many songbirds are under a strong selection pressure to migrate quickly. Thus, the ability to effectively refuel at stopover sites is likely a key driver of individual success during migration and possibly in subsequent stages of the annual cycle.

Despite the hypothesized importance of refuelling at stopover sites for subsequent migration, there is no direct evidence that fuel loads acquired during particular stopovers carry-over to influence the overall pace of migration. This is primarily due to the difficulty of measuring individual behaviour at stopover sites (e.g. mass gain, departure) and then tracking their subsequent migration, which may take place over thousands of kilometres. However, the advent of automated radio-telemetry systems has increased the scale at which detections of animal movements are possible^33^, without having to rely on individuals being recaptured to acquire movement data over vast areas.

**Figure 1:**
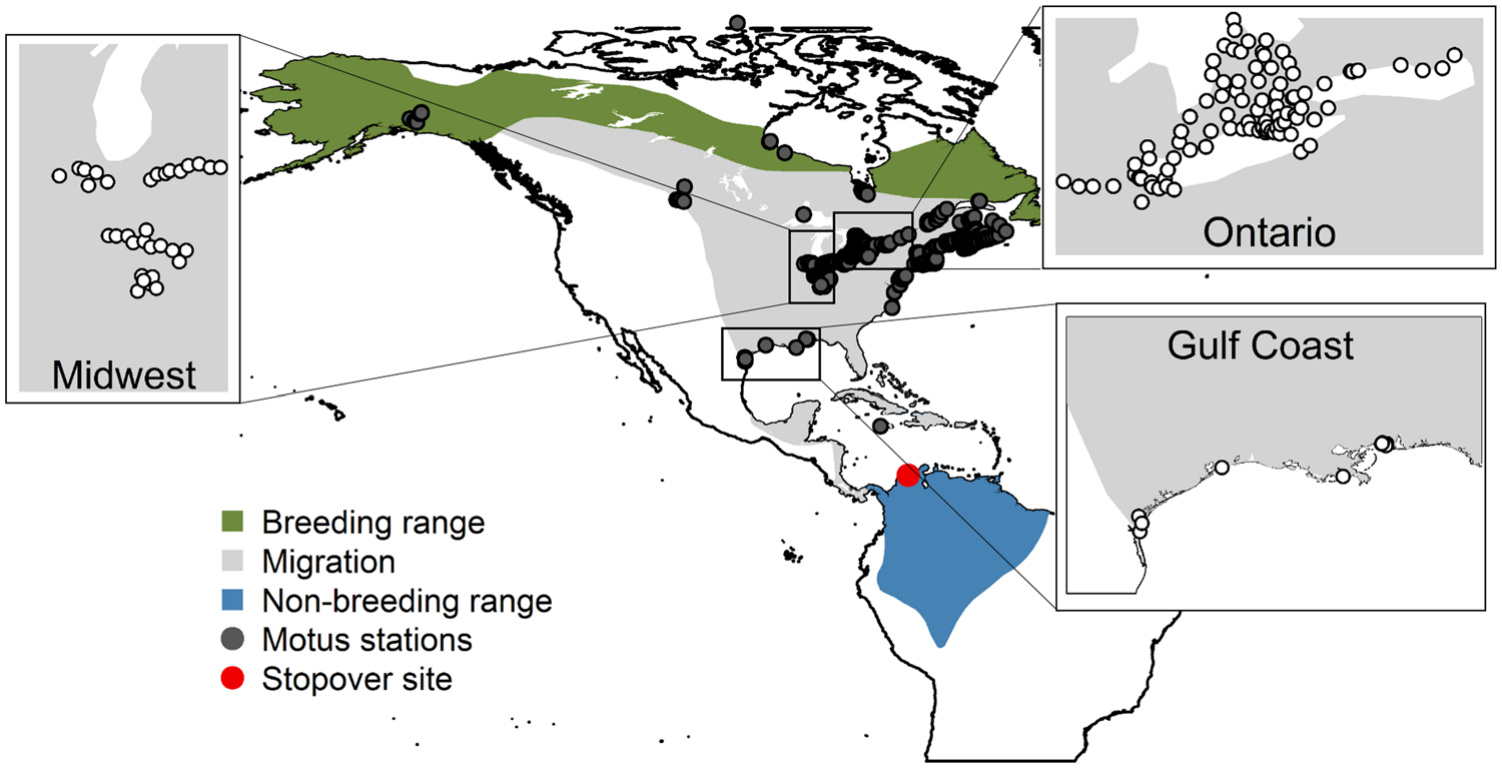
Known distribution map of the Grey-cheeked Thrush^36^, highlighting the spring stopover site in northern Colombia. The three zoomed panels show the regions with most of the detections of migrating Grey-cheeked Thrush in North America, following their departure from Colombia. Dots represent the automated receiving stations that were operational during 2015 and 2016. Map generated using BirdLife International and Handbook of the Birds of the World (2016) Bird species distribution maps of the world. Version 6.0. Available at http://datazone.birdlife.org/species/requestdis.

We combine field monitoring of stopover behaviour with direct tracking using an intercontinental array of automated telemetry stations to test predictions of optimal migration theory and to quantify the effects that departure fuel loads acquired at a spring stopover site in northern Colombia have on the subsequent pace of migration of a long-distance migratory songbird, the Grey-cheeked Thrush (*Catharus minimus*). The Grey-cheeked Thrush travels more than 10,000 km annually between breeding grounds in the boreal region of Canada and Alaska and wintering grounds in the northern Amazon Basin (Fig. 1)^34–36^. During spring migration, many Grey-cheeked Thrush make a stopover in northern Colombia prior to crossing the Caribbean en route to their North American breeding grounds^37, 38^. Based on rates of mass gain and estimated fuel loads, individuals departing from this stopover site are thought to be capable of long-distance flights (>2500 km) without needing to refuel^37^, suggesting this species optimizes its stopover behaviour to minimize the overall time of migration. Whether Grey-cheeked Thrush actually carry out these long over-water flights after their spring stopover in Colombia, however, is unknown.

Here, we examine the hypothesis that this species is a time-minimizer by testing the predictions that (1) fuel loads are steeply and positively correlated with fuel deposition rates, (2) stopover duration is adjusted in response to local fuelling rates, (3) birds take direct routes across a large water barrier, and (4) the pace of intercontinental migration is positively influenced by refuelling rates at the stopover site. More broadly, we ask whether fuel loads acquired at a single spring stopover site influence the pace of intercontinental migration in this songbird.

## Results

### Apparent stopover duration of radio-tagged and untagged birds

**Figure 2:**
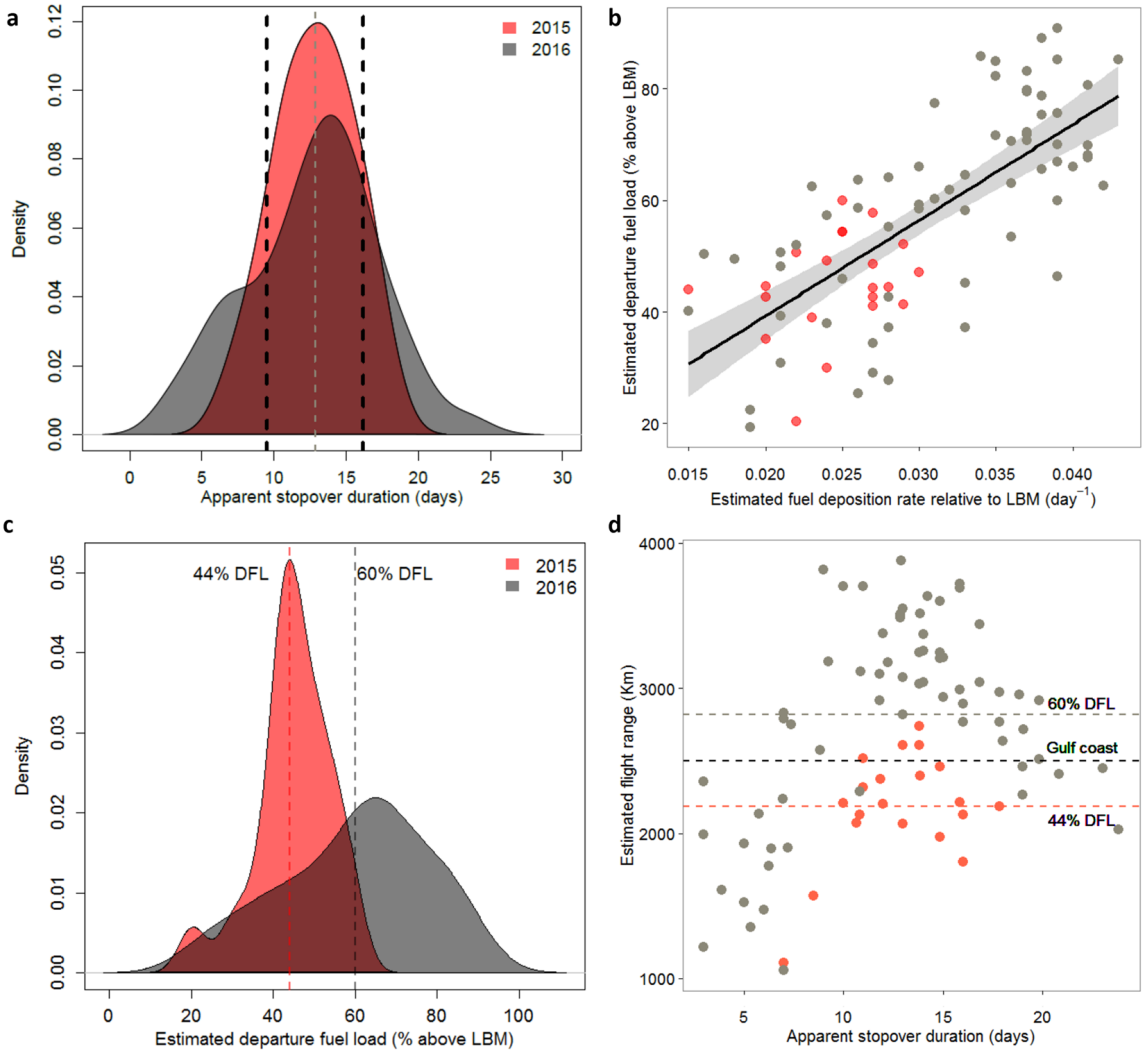
Evidence for time-minimizing migratory strategy in the Grey-cheeked Thrush. (**a**). Despite yearly differences in fuelling rates, apparent spring stopover duration in the Sierra Nevada de Santa Marta, Colombia, did not differ between years. Shaded polygons represent the probability density of estimated stopovers of tagged individuals (mean 2015 = 12.8 and 2016 = 12.6, area under the curves = 1), and vertical lines show the mean ± se of stopover durations estimated from all recaptured birds using capture-recapture models (12.84 ± 3.3 days for both years). (**b**) As expected for time-minimizers, tagged Grey-cheeked thrush showed a strong positive correlation between departure fuel load and daily fuel deposition rate. The steep slope of this relationship as well as a lack of an asymptote suggests this is not a total energy cost minimizing strategy. (**c**) Slower fuelling rates in 2015 resulted in significantly lower departure fuel loads (DFL) compared with 2016 as shown by the dotted lines. (**d**) As a consequence, birds in 2015 were predicted to have shorter mean potential flight ranges (~2200 km, red dotted line) than birds in 2016 (~2800 km, grey dotted line). Peak departure fuel load and flight ranges in both years were achieved within a 10–16 d period at the stopover site. Assuming no wind assistance, more Grey-cheeked Thrush leaving northern Colombia in 2016 were expected to be able to fly directly to the Gulf-coast (black dotted line) and beyond.

Of 888 Grey-cheeked Thrush captured in the Sierra Nevada de Santa Marta, Colombia (479 in 2015 and 409 in 2016), 53 individuals (6%) were recaptured on one or several occasions (21 in 2015 and 32 in 2016), including 11 of 133 (8%) radio-tagged birds. Most individuals arrived with low body mass and low fat scores (Supplementary Fig. S1a–c), indicating depleted energy reserves. Apparent stopover duration of radio-tagged birds was 12.7 days and did not differ between years, although the variation in stopover duration was wider in 2016 (Fig. 2a; 2015: mean ± SD = 12.8 ± 2.8 d, *n* = *23*; 2016: 12.6 ± 5.0 d, *n* = *72*). Stopover duration of untagged birds, estimated using capture-recapture models, was 12.8 ± 3.3 d for 2015 and 12.7 ± 3.1 d for 2016 (Fig. 2a). The model receiving the highest AICc support had a 0.53 weight (Table 1, Fig. 2a), and predicted a negative effect of date on the probability of remaining at the stopover site (ɸ, ‘survival’) and on the probability of being present at the study site prior to capture (ɣ, ‘seniority’), implying a decrease in stopover duration as the season advanced (Supplementary Fig. S2a). Estimates of stopover duration did not differ between years when averaging the first five models which were within 6 AIC values^39^ (12.8 ± 2.4 days for 2015, and 12.7 ± 2.4 days for 2016).

**Table 1:** 20 Cormack-Jolly-Seber models were evaluated to determine total stopover duration of untagged Grey-cheeked Thrush during the spring migrations of 2015 and 2016 in northern Colombia. Of these, the top model had a 0.53 weight and was used to obtain the probability estimates of ɸ (survival), ɣ (seniority) and *p* (recapture). See Table S2 for the complete list of models.

Models of stopover duration	Parameters	AICc	Δ AICc	*Wi*
**ɸ(~Date)p(~1) ɣ (~Date)**	**5**	**836.67**	**0.00**	**0.53**
ɸ(~Date)p(~1) ɣ (~Date + mass)	6	838.90	2.23	0.17
ɸ (~year + Date)p(~1) ɣ (~Date)	7	840.99	4.32	0.06
ɸ (~Date)p(~1) ɣ (~Date + year)	7	841.18	4.51	0.06
ɸ (~year)p(~1) ɣ (~Date)	6	841.38	4.71	0.05
ɸ (~year + mass + Date)p(~1) ɣ (~Date)	8	843.30	6.63	0.02

### Fuel deposition rate, departure fuel load and potential flight range

We evaluated eight generalized additive models to describe change in body mass of recaptured birds as a function of days since first capture (Table S3). A model including an effect of date of first capture and of year received very strong support (Fig. S1b. AICc = 409.5, *wi* = 0.86), implying that change in body mass was smaller for birds captured later in the season and was slower in 2015 than in 2016. There was little support for an effect of either age or whether birds carried a radio-tag.

All tagged individuals making a stopover of more than 48 hours at our site gained mass before their estimated departure. As predicted for time-minimizers and for total-energy-minimizers, there was a strong positive correlation between predicted values of departure fuel load and fuel deposition rate (Fig. 2b, β_FDR_ = 18.34, R^2^ = 0.69 P < 0.001), suggesting that birds which accumulated fuel at higher rates were also heavier upon departure from the stopover site in northern Colombia. However, the steep slope (β_FDR_ = 18.34) and the lack of an asymptote in the relationship between FDR and DFL at high fuelling rates is consistent with a time-minimizing strategy and does not fit the expectation for a total-energy-minimizing strategy. Also consistent with the hypothesis that migrants are time-minimizers were the lower departure fuel loads in 2015, likely a consequence of the lower fuelling rates in that year compared to 2016 (Fig. 2c). In 2015, birds left with a mean fuel load equivalent to 44% of lean body mass, whereas in 2016 they left with a mean fuel load of 60% (Fig. 2c). These fuel load differences resulted in mean (±SE) flight-range estimates of 2200 ± 600 km in 2015 (range = 1000–2800 km) versus 2800 ± 1200 km in 2016 (range = 1000–4000 km; Fig. 2d), implying that, without wind assistance, fewer birds in 2015 (c. 25%) than in 2016 (c. 75%) would have been able to fly directly from Colombia across the Caribbean Sea to the U.S. Gulf Coast (~2500 km) without making additional re-fuelling stopovers. Flight range estimates accounting for the effects of drag caused by radio-tags suggested a minimal difference in range due to drag (100–500 m) when estimated in the program Flight^40^.

### Pace of intercontinental migration

**Figure 3:**
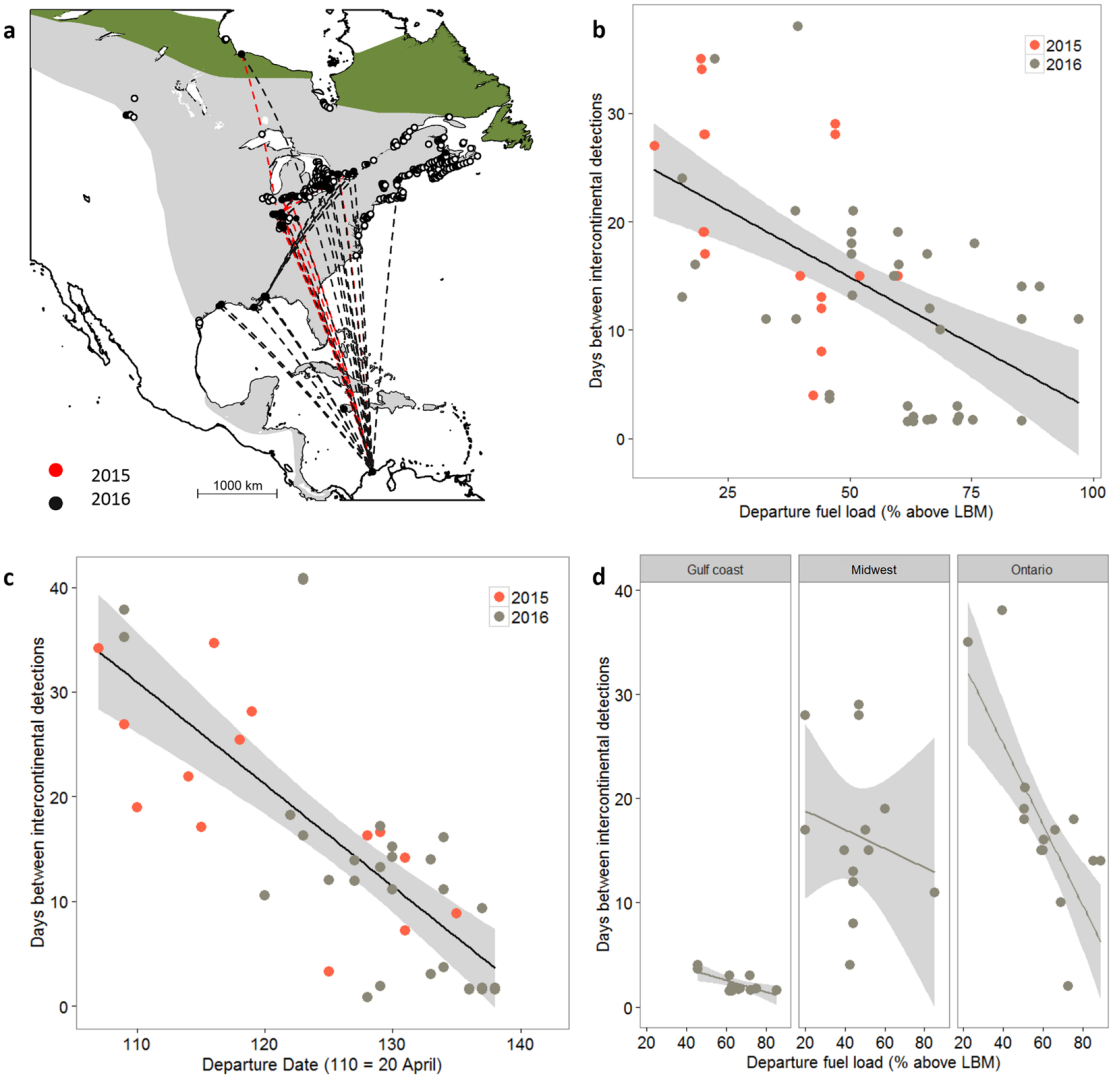
Evidence for an effect of departure fuel loads and departure date on the subsequent pace of migration. (**a**) 43 radio-tagged Grey-cheeked Thrush were detected in North America by automated receivers. Dashed lines connect the great arc distance between detections but they should not be interpreted as flight trajectories. 30 birds were detected on multiple occasions, all of which show a strong north-east shift in direction after first detection. Map generated using ‘maptools’ version 0.8–39 in R^90, 104^ (https://cran.r-project.org/package = maptools). (**b**,**c**) We found a strong negative relationship between departure fuel load (**b**) and departure date (**c**) on the pace of migration, measured by the number of days elapsed between intercontinental detections of Grey-cheeked Thrush departing from northern Colombia. Shaded areas represent 95% confidence intervals. (**d**) The best model supported a significant effect of the geographic region where the detections occurred as determinants of the pace of migration after accounting for the effect of departure fuel load (Gulf coast: n = 12, Midwest: n = 13, Ontario: n = 14). The magnitude of the region effect increased with increasing distance from Colombia, suggesting a negative carry-over effect on the pace of migration after the Caribbean-Gulf crossing.

Radio-tagged Grey-cheeked Thrush departed our study site in northern Colombia between 18 April and 21 May, with the peak of departures occurring on 6 May ± 3 d in both years. Of 133 radio-tagged birds, 43 (32%) were detected at least once by the automated array of receivers at various points in North America en route to their breeding grounds (n = 14 in 2015 and n = 29 in 2016; Fig. 3a.), with 30 of these individuals being detected on more than one occasion. There were no differences in detection probabilities of birds carrying ratio-tags of different weight or burst-rate configuration. Detections in North America were concentrated within three main areas: The Gulf Coast, the Midwest (states of Ohio and Indiana), and southern Ontario. Two birds were detected in Hudson Bay, presumably on, or very close to their breeding grounds and >5000 km from their tagging site in Colombia (Fig. 3a).

Based on the time elapsed (range 20–79 hours, Table S4) and the distance covered between detections (870–3500 km, Table S4), we inferred that 11 birds made direct flights from our site in Colombia to either Jamaica (n = 1), the Gulf Coast (n = 8), the Midwest (n = 1), or southern Ontario (n = 1; Fig. S2a). The speed of migration of these birds, estimated assuming they flew continuously (40–75 km/h, Table S4), was within or above the range of flight speeds (max 65.8 km/h) known for migrating *Catharus* thrushes measured directly in North America^41^ (Fig. S2b). Furthermore, the distances these birds flew were within estimated flight ranges (Fig. 2d), supporting the inference that they likely did not stop to refuel between their departure and first detection in North America. The detection in Jamaica consisted of several hits over a 5-min period at a single receiver, suggesting that the bird continued its flight to North America. The fastest bird was detected in southern Ontario only 46 hours after its departure from Colombia, which means it travelled at a minimum speed of 76 km/h (Fig. S2b, Table S4), setting a known flight speed record for this species. Although local wind conditions were calm on the night of departure of this bird (data from a weather station at the local airport), it probably encountered favourable tail winds once it entered the Gulf of Mexico (https://earth.nullschool.net/#2016/05/09/1200Z/wind/surface/level/orthographic = −87.49,24.85,787/loc = −76.024,13.759), which would have increased its average flight speed.

Finally, as predicted for time-minimizers, we found a negative correlation between the pace of northward migration and the estimated departure fuel loads of birds leaving Colombia, with a maximum difference of 30 d of travelling time between the birds with the lowest and highest departure fuel loads in our sample (Fig. 3b. β_DFL_ = −27.57, R^2^ = 0.29, P < 0.001). Furthermore, departure date from Colombia also had a strong negative effect on the pace of migration (Fig. 3c. β_Depart_ = −0.76, R^2^ = 0.56, P < 0.001): birds that departed later in the season reached North America faster than those departing earlier. A linear mixed-effects model, with a 0.99 weight, predicted significant effects of departure fuel load, date of departure, and geographic region on the pace of migration (Fig. 3d, Table S5). The slope of the relationship increased as birds were detected further away from Colombia (Fig. 3c), suggesting that leaner birds had to stopover again after the Caribbean-Gulf crossing, further delaying their northwards advance.

## Discussion

Our results suggest that a single stopover can have a substantial effect on the pace of migration of a long-distance migratory songbird. Variation in the fuel loads and departure date from a spring stopover site in northern Colombia resulted in a difference of up to 30 d in travelling time for Grey-cheeked Thrush that were subsequently detected up to 4500 km away in southern Ontario, Canada (Fig. 3b and c). Although there are other factors that likely contributed to variation in the pace of migration, such as wind conditions en route^42, 43^ and the quality of stopover sites encountered along the way, we provide convincing evidence of how this stopover site played an important role in the subsequent migration of Grey-cheeked Thrush. Our results also suggest that this species behaves according to predictions of the time-minimization hypothesis, by (1) showing a steep positive relationship between departure fuel loads and fuel deposition rate without signs of levelling off at high fuelling rates (Fig. 2b), (2) showing a strong negative relationship between pace of migration and departure date where later birds migrated faster (Fig. 3c), and (3) taking the shortest routes reflected in the direct flights from Colombia to the Gulf Coast and beyond (Fig. 2c and d, Fig. S2)^18, 44^. The time cost incurred by birds that leave Colombia in poor condition may have detrimental fitness consequences if it results in a later arrival to the breeding grounds^45^, especially in light of evidence that, in other songbirds, a 15 d difference in arrival on the breeding grounds may result in significant variation in reproductive success^14^. Our results emphasize the need to identify major fuelling regions for migratory birds, especially in the Neotropics where studies are rare, and to include stopover periods in analyses of migratory connectivity and population dynamics because these sites could be acting as bottlenecks for migratory species^46–48^.

Monitoring the stopover behaviour and intercontinental movements of radio-tagged birds allowed us to test predictions of optimal migration theory^18^. The strong positive correlation between departure fuel load and fuel deposition rate, plus a lack of an asymptote at high fuelling rates suggest that Grey-cheeked Thrush are time-minimizers^17, 18^. Although distinguishing between time-minimization and total energy-minimization can be difficult^18^, the magnitude of the barrier to be crossed during migration (~2500 km), the fuel loads observed in this study and the slope of the relationship with fuelling rates were comparable to those of other time-minimizing songbirds preparing to cross the Sahara desert^49, 50^ and the long flights characteristic of time-minimizing shorebirds^44, 51^. However, because we did not directly measure individual fuel deposition rates (i.e. we estimated them based on the mean body mass change in the population), a direct comparison of the values found here and those measured for other species, should be made with caution. Regardless, our results and those of others^52–54^ show that many songbirds make few but relatively long stopovers that are followed by long flights, a strategy also characteristic of shorebirds^21, 51^. However, unlike most shorebirds, important refuelling regions, particularly in the Neotropics^55^, have not been identified for most songbirds. Identifying and conserving key refuelling regions will be paramount for the successful conservation of species like the Grey-cheeked Thrush, which complete a large proportion of their migration route within the Neotropics.

From both radio-tagged birds and from recapture probabilities of all banded birds, we found that, despite differences in fuelling rates between years (Fig. 2c), mean stopover duration was similar (Fig. 2a). This is not consistent with the predictions of time-minimization, where birds are expected to adjust stopover duration to local and expected fuelling rates^18, 56^. However, variation in stopover duration was larger during 2016, the year with higher fuelling rates (Fig. 2a) and presumably more resources. The higher variation in 2016 suggests that during years with plentiful resources a wider variety of migration strategies can occur^57, 58^. Some studies have provided evidence of phenotypic plasticity in migratory strategies but also that harsh conditions can restrict the range of behaviours expressed by individuals^58–60^. In years with benign conditions some individuals may adjust to a shorter stopover duration, a mechanism allowing them to achieve time-minimization^18, 61^, while others may extend their stay and then fly faster and cover a longer distance^17^, hence achieving time-minimization as well. The modal peak in stopover duration at 13 days during both years suggests that there is an optimal duration that most individuals adhered to at this site. Migratory strategies in long-distance migrants are thought to be under tighter endogenous control when compared to short distance-migrants, giving rise to a centralization of phenotypes^58, 62^ and this may well be the case for the stopover duration of Grey-cheeked Thrush in northern Colombia. Whether birds are behaving according to a ‘constant stopover duration’ rule of thumb to approach time-minimization^61^ or whether there is a mixture of strategies within the population will have to be assessed by future studies considering more stopover sites throughout the species migratory route.

Departure fuel load as well as departure date from Colombia had a significant effect on the pace of migration of Grey-cheeked Thrush (Fig. 3b,c). This could be a result of higher time constraints later in the season, consistent with time-minimization, or of the use of various strategies within the population, where some birds minimize time while others migrate at a more leisurely pace. There are examples in the literature where, like in our study, birds that depart later, do so with higher fuel loads and migrate faster^57, 63^, as well as evidence to the contrary where late departures arrive later to their destination^53, 64^. A recent study found that individuals can modify the pace of migration in response to environmental conditions, and that they can respond differently to the same conditions depending on the stage of their migration^62^. Although we lack the data to tease apart these possibilities, all the other sources of evidence from this study point towards a time-minimization strategy for the Grey-cheeked Thrush.

Not all stopover sites are expected to provide the same services for migrants^65, 66^. Stopover sites located adjacent to barriers, where migrants accumulate large fuel reserves needed for long flights, are likely critical for determining the pace and success of migration^66–69^. Although birds with relatively small fuel loads appeared to take substantially longer to fly between Colombia and North America, a lack of knowledge of the final breeding destinations of these individuals limits our ability to infer possible timing-related carry-over effects on the breeding grounds relative to reproductive performance. Birds may make up for lost time during migration^70^ or may still be successful even when arriving late to the breeding grounds^71^. However, most evidence suggests substantial negative carry-over effects on fitness related to the timing of arrival on the breeding grounds^69, 72–74^ even in cases where delays were of smaller magnitudes than those reported here^14, 53^.

Aside from showing the extreme importance of a single stopover site for determining the pace of migration, our study also reveals several novel aspects of Grey-cheeked Thrush migration. For instance, our data show that individuals actually cross the Caribbean Sea and the Gulf of Mexico directly without the need to refuel, as had been hypothesized previously^37^ but never confirmed. Also, our multiple intercontinental detections of radio-tagged birds provide the first direct evidence of a spring migration route for at least a portion of the eastern population of Grey-cheeked Thrush, whereby birds cross the Caribbean, then appear to make a north-east turn after reaching the Gulf Coast, and head towards the Great Lakes, where presumably some continue to the eastern portion of their breeding range (Fig. 3a). Radio-tagged Grey-cheeked Thrush in this study did not appear to make multi-day stopovers on the Gulf Coast; instead, detections from automated stations suggested that birds overflew Motus stations along the coast and continued further inland with some individuals continuing to the Midwest or southern Ontario. We did not find evidence for differences in fuel loads or migration route between adults and second-year birds, as has been reported for a closely related species, the Wood Thrush (*Hylocichla mustelina*)^53^.

The use of a collaborative automated telemetry network in this study was critical for monitoring the stopover behaviour of individuals and the subsequent pace of their migration^33^. Nearly all radio-tagged birds were followed until departure from our study site, compared with only a 6% recapture probability of banded birds at our site. Furthermore the percentage of radio-tagged individuals subsequently detected in North America (32%) was much higher than the recovery rate of most banded songbirds (close to zero) and is similar to recoveries of birds carrying geolocators^53^. This recovery rate is remarkable considering that coverage by automated telemetry stations can still be substantially improved and highlights the potential of automated telemetry to study species movements without the need to recapture them. As currently configured, 0.98 g radio-tags last for 12 months and, therefore, have the potential of providing precise spatial information on the annual movements of migratory animals that weigh as little as 20 g. Improvements in technology will likely reduce the cost, weight and increase the life-span of radio-transmitters even further. The usefulness of this technology for broad-scale migratory applications, however, is dependent on the availability of a large network of stations that can detect radio tags. Increasing the coverage of the Motus network^33^ as well as reducing the costs of tags and equipment are promising ways to gather movement data for the full annual cycle of migratory animals^33^.

Our study provides compelling evidence that a single stopover can significantly influence the pace of migration, with important implications for the conservation of migratory songbirds^75^. Migration is still the least studied period of the annual cycle of most migratory birds despite accumulating evidence suggesting that it is a critical period that can impact population dynamics^5, 48, 69, 76^ (but see ref. 77). Identifying critical stopover sites for declining migratory bird populations throughout their migration routes is therefore a high conservation priority. Similar to how Delaware Bay^51, 72^ and the Yellow Sea^51, 78^ are critical for shorebirds, the Sierra Nevada de Santa Marta in northern Colombia is likely important for the Grey-cheeked Thrush and potentially for up to 40 other migratory songbird species that regularly use this stopover site^79^. The value of the Santa Marta forests to migratory birds adds to this site’s recognition as one of the most irreplaceable regions on the planet due to its high endemism and unique biological and cultural diversity^80^. There is no doubt that other critical regions for migratory songbirds exist throughout the world^65^. Our work illustrates that by combining the latest technological advances for tracking birds with on-the-ground field studies, researchers are likely to continue unravelling the mysteries of migration, while rapidly identifying the sites most crucial for the long-term persistence of migratory species.

## Methods

### Spring migration monitoring

During the spring migrations of 2015 and 2016 (April – May), we banded 888 Grey-cheeked Thrush captured in constant-effort monitoring stations (30 12-m mist-nets) in the Sierra Nevada de Santa Marta, Colombia (11.122° N, −74.087° W, mean elevation 1100 m). Stations were run for six hours daily starting at dawn, weather permitting, and we recorded the age^81^, body mass and wing length of all individuals captured and recaptured during the entire spring passage^37, 38^. In addition, 133 individuals were affixed with Lotek (NTQB-2, -3-2, and -4-2, Newmarket, ON, Canada) digitally coded radio transmitters (36 in 2015 and 97 in 2016) weighing either 0.98 g (n = 47), 0.67 g (n = 53) or 0.35 g (n = 33) and programmed at a single frequency (166.380 MHz). Battery size determines the weight and, together with burst-rate, defines the life of a radio-tag which ranged from 54 to 306 d in our study (see supplementary Table S1 for details). We attached radio-transmitters using leg-loop harnesses adjusted for body size^82, 83^. The mass of the heaviest transmitter and harness was less than 4% of the estimated lean body mass of Grey-cheeked Thrush migrating through the study area, which is approximately 26 g^37^.

### Apparent stopover duration of radio-tagged birds

To determine apparent stopover duration and departure date of radio-tagged birds, we used telemetry data from two automated receiving stations. Each station consisted of three nine-element Yagi antennas (Laird Technologies, PLC-1669) and a SensorGnome receiver (https://www.sensorgnome.org/). Stations were installed at high vantage points (11.123°, −74.089°; and 11.123°, −74.093°), and antennas were oriented to maximize local detections and to ensure clear departure signals from northward departing birds^84^. Each antenna has a ~12 km detection range under ideal conditions (i.e. birds in flight and within range of the antenna)^85^. Clear departure signals consist of a series of continuous detections that progressively increase and then decrease in signal strength as a bird flies towards, over, and then away from the station in a given direction, and which produces a characteristic peak in signal strength^33, 86^. A bird was considered to have departed when such signals coincided with the last day that it was detected by automated receivers.

Apparent stopover duration was estimated as the number of days from first capture (and radio-tagging) to departure. We refer to this as ‘apparent’ rather than actual stopover because we are uncertain when captured birds first arrived at the site. We considered apparent stopover to be reliable (n = 95) when we had detections for more than two days after first capture, combined with a departure signal from the automated receivers between 18:00 and 21:00 hours, when *Catharus* thrushes are expected to initiate migration^86–88^. For 38 birds, we considered our estimate of stopover duration to be unreliable either because A) birds moved beyond the reach of our local stations within 48 hours of radio-tagging (n = 25), B) they disappeared at times of the day or night suggesting landscape movements other than migratory departures^85, 89^ (n = 11), or C) birds had not yet left the study site prior to May 15 when local receiving stations were dismantled (n = 2, only in 2015). During both years, receiving stations were operational from April 4, which is before the main Grey-cheeked Thrush spring passage begins at our study site^37, 38^. In 2015, receiving stations were dismantled on May 15 but in 2016 they ran until all radio-tagged birds had left. Generalized linear models were used to evaluate whether apparent stopover duration varied as a function of date of capture and of mass on first capture^90^.

### Stopover duration of birds that were not radio-tagged

We estimated the stopover duration of recaptured birds using Cormack–Jolly–Seber (CJS) models to estimate ‘total stopover’^37, 91^. Briefly, this involves constructing capture histories for each bird where every day of mist netting is considered a capture occasion, and each occasion receives a score of either 1 (bird captured) or 0 (not captured). This method estimates the survival probability (ɸ), which, in the context of this analysis, is the probability of staying at the stopover site after being caught; the seniority probability (ɣ), which is the probability that an individual was present at the site before it was captured; and the probability of recapturing an individual (*p*)^91^. A life-expectancy equation transforms these probabilities into time intervals (days in our case) whereby total stopover is estimated as: TS = (−1/ln(ɸ)) + (−1/ln(ɣ))^91^. Analyses were carried out using package ‘RMark’ in R^92, 93^. We evaluated models in which the probability of a bird remaining at the stopover site after capture (ɸ) was constant or varied as a function of year, date and mass on first capture. We assumed the probability of being present at the site before capture (ɣ) varied with date, as the phenology of migration is bell shaped^37^. We also evaluated the effect of year and mass on first capture on ɣ. Probability of recapture (*p*) was assumed to remain constant throughout the season. This resulted in 20 possible models that included all the combinations of covariates associated with ɸ and ɣ (Table S2). Model fit and selection was carried out under a likelihood framework based on AICc values^92–94^, and the best model was used to estimate total stopover duration.

### Fuel deposition rate, departure fuel loads and potential flight range

Fuel deposition rate (*FDR*) is the daily rate at which individuals accumulate fuel during a stopover^21^. Because 95% of the fuel accumulated by migrants is fat and only 5% is protein^95^ we followed other studies^18, 44, 49^, in assuming all the weight put up by migrants during stopover is made up of fat. Fuel deposition rate is expressed as mass of fuel accumulated daily relative to lean body mass (*LBM*), which is the mass of birds with no visible fat reserves. Departure fuel load (*DFL*) is the total mass of fuel, relative to lean body mass, with which an individual departs from a stopover site; this variable will determine the potential flight range (i.e., the distance a bird can fly after its stopover)^67^.

We estimated fuel deposition rate from all recaptured birds (radio-tagged and untagged) at our site by calculating their daily rate of change in body mass between capture occasions. We used generalized additive models^96^ to predict change in body mass as a function of days since first capture^37, 68, 97^, and evaluated the effect of date of first capture, year, age, mass on first capture and presence or absence of a radio-tag. We also included a random effect of individual in the model to account for birds that were recaptured more than once. These models were run using package ‘mgcv’ in R^98^. The resulting best-fit model was used to predict the change in body mass of all radio-tagged birds which were not recaptured (N = 122), by using their initial mass and their apparent stopover duration estimated from the receiving stations. These estimates of change in body mass (*Δ mass*) were subsequently used to calculate fuel deposition rate using the equation *FDR* = (Δ*mass*/*St*)/*LBM* ^99^, where *St* is the apparent stopover duration. Lean body mass was estimated using a regression of body mass and wing length of all birds captured with a fat score of zero (n = 135)^37^, which gave rise to the linear equation *LBM* = 0.33 × *wing length − *4.63, (R^2^ = 0.25, P < 0.001). Departure fuel load was estimated using the equation *DFL* = ((*mass*_*t*0_ + *Δ mass*)−*LBM*)/*LBM* ^25, 67^, where *mass*_*t0*_ is the mass of the bird on first capture. Potential flight range depends on departure fuel load and on the mean airspeed of flight *U* (~60 km/h for the Grey-cheeked Thrush)^41^ and can be estimated using the equation *Flight range* = 100 × *U *× In (1 + *DFL*)^67^. Finally, we used Program Flight^40^ to estimate flight range accounting for the drag of radio-tags^100^. Using a mean wing span of Grey-cheeked Thrush (0.307 m, n = 5^37^), we estimated flight ranges by increasing body drag to 2.0^100^.

### Pace of intercontinental migration

To track individual thrushes departing from our stopover site, we used the Motus Wildlife Tracking System (http://motus.org/), an international collaborative research network maintaining coordinated automated receiving stations throughout the Americas^33^. Between April and July 2015–2016, more than 300 automated receiving stations in North America were operational (Fig. 1) and able to record the unique ID of radio-tags linked to the Motus frequency (166.380 MgHz). Not all stations were operational during both years, with stations on the northern coast of the Gulf of Mexico becoming operational only during 2016, increased coverage in parts of the North-east during 2016, while the Midwest had approximately a 50% reduction of active stations in 2016. Detections of radio-tagged Grey-cheeked Thrush were filtered to include only true signals distinguishable by at least three consecutive signal bursts at the tag’s designated interval^101^. For all the individuals detected in North America, we obtained the location and exact time of detection, enabling us to estimate the pace of migration (which includes periods of both flight and stopover between detections). We estimated the geographic great-arc distances between intercontinental detections using package ‘geosphere’ in R^102^.

We assessed whether the time elapsed between intercontinental detections varied as a function of departure fuel load and of departure date from Colombia. Because most of the detections in North America were concentrated within three main geographical regions (the Gulf coast, the Midwest and southern Ontario, Fig. 1), we evaluated whether the pace of migration varied among birds detected within those three regions. We ran linear mixed-effects models including region as a covariate and a random effect of bird individual, to account for birds detected on multiple occasions. Models were fit with package ‘lme4’ in R^103^.

### Animal handling and ethics

All animal handling and tagging procedures were carried out in accordance to international standards and were approved by the animal care and ethics committee of the Universidad de Los Andes - CICUAL (Acta 293, C.FUA_14-016). Research permits were issued by *Agencia Nacional de Licencias Ambientales* (Res. 0597).

### Data availability

All data are available by request through the Motus Wildlife Tracking System (motus.org).

## Electronic supplementary material

Supplementary information
