# Supplementary material for: Fuel loads acquired at a stopover site influence the pace of intercontinental migration in a boreal songbird
Source: Sci Rep. 2017 Jun 13;7:3405. doi: 10.1038/s41598-017-03503-4 (PMC5469819; doi:10.1038/s41598-017-03503-4)
Supplement: Supplementary file 1 — Supplementary information [file 41598_2017_3503_MOESM1_ESM.pdf]

Fuel loads acquired at a stopover site influence the pace of intercontinental migration in a boreal songbird

Supplementary Information

Camila Gómez [*1,2], Nicholas J. Bayly [2], D. Ryan Norris [3], Stuart A. Mackenzie [4], Kenneth V. Rosenberg [5], Philip D. Taylor [4,6], Keith A. Hobson [7,8], Carlos Daniel Cadena [1]

[1] Laboratorio de Biología Evolutiva de Vertebrados, Departamento de Ciencias Biológicas, Universidad de los Andes, Bogotá, Colombia; [2] SELVA: Investigación para la conservación en el Neotropico, Bogotá, Colombia; [3] Department of Integrative Biology, University of Guelph, Guelph, Ontario, Canada; [4] Bird Studies Canada, Port Rowan, Ontario, Canada; [5] Cornell Lab of Ornithology, Ithaca, New York, USA; 6 Acadia University, Wolfville, Nova Scotia, Canada; [7] Environment and Climate Change Canada, Saskatoon, Saskatchewan, Canada; [8] Department of Biology, University of Western Ontario, London, Ontario, Canada;

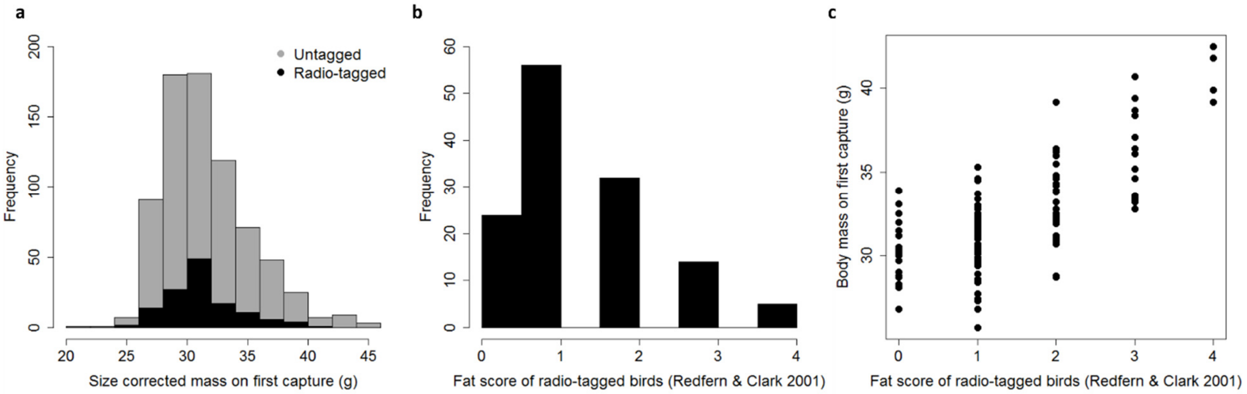

Figure S1. Body condition of Grey-cheeked Thrush upon first capture at our study site in northern Colombia. Body mass on first capture (**a**) as well as fat score [1] (**b**) showed a skewed distribution towards lower values suggesting most birds arrived with minimal energy reserves. Heavier birds were likely captured several days after arrival. There is a tight correlation between fat score and body mass (**c**) suggesting both are good indicators of body condition.

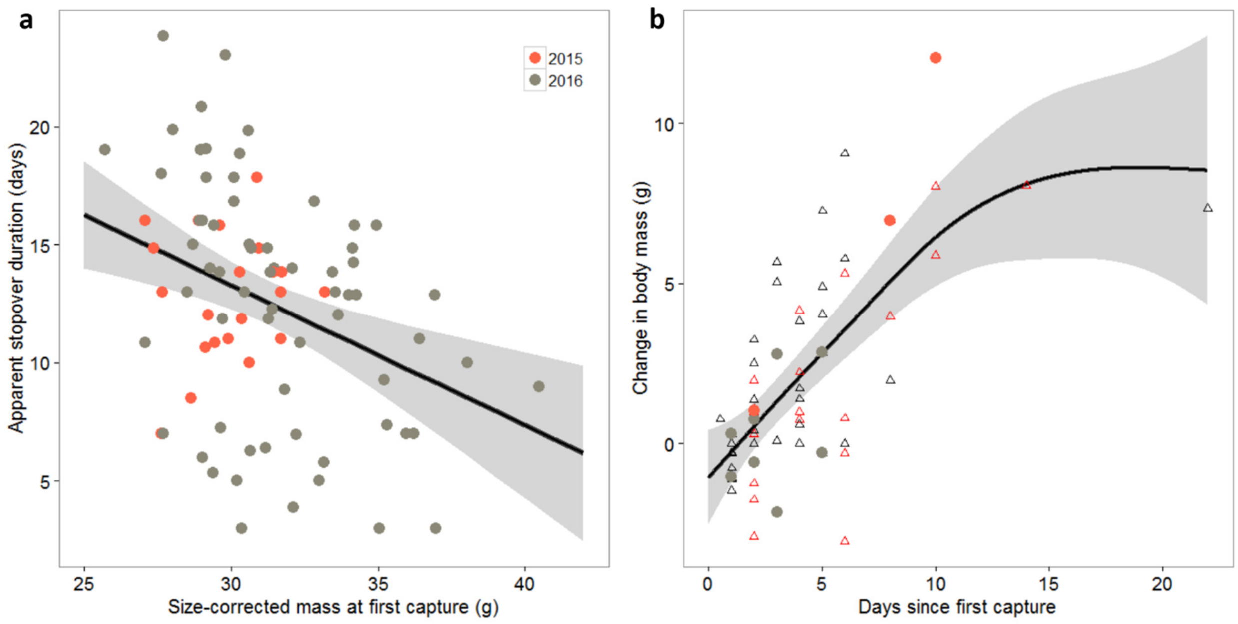

Figure S2. a. Mass on first capture significantly affected the stopover duration of tagged birds, with lean birds apparently stopping over for longer than fat birds ($R^2 = 0.17$, $P < 0.001$). **b.** Change in body mass as a function of days since first capture did not differ between tagged (circles) and untagged (triangles) birds. The best fit model included an effect of date of first capture and an interaction with days since first capture and year (AICc = 409.5, *wi* = 0.86).

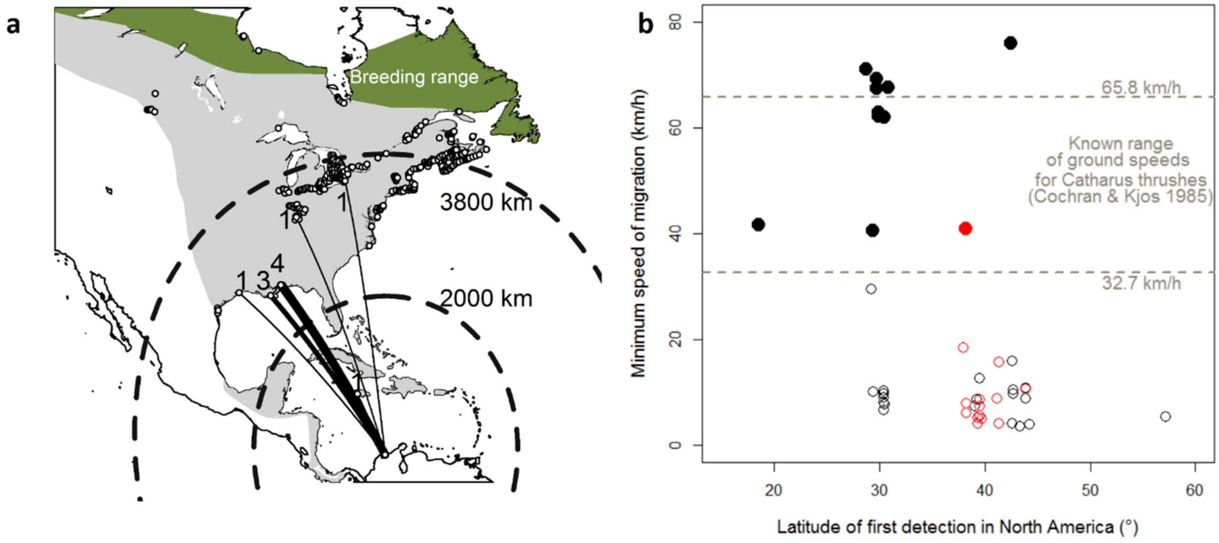

Figure S3. a. 11 birds made direct flights from Colombia to Jamaica (1), the Gulf coast (8), Indiana (1) or southern Ontario (1). These areas are within the predicted flight ranges based on departure fuel loads from Colombia. **b.** Birds that made direct flights (filled symbols) had minimum migration speeds (including flight and resting) which were either within or above those known for flying *Catharus* thrushes, suggesting they did not make fuelling stopovers between detections (red = 2015, black = 2016). Open symbols show the rest of the birds first detected in North America, which were below known flying speeds. Map generated using BirdLife International and Handbook of the Birds of the World (2016) Bird species distribution maps of the world. Version 6.0. Available at http://datazone.birdlife.org/species/requestdis.

Table S1. Estimated radio-tag lifespan according to mass (battery size) and burst rate interval. In parenthesis are shown the numbers of radio-tags with each configuration used in this study.

	Estimated 80% tag lifespan in days		
	Tag Model		
**Burst rate**	**0.35 g** (NTQB-2)	**0.67 g** (NTQB-3-2)	**0.98 g** (NTQB-4-2)
6 sec.		90.85 (n = 23)	
11 sec.	54.29 (n = 29)	130.24 (n = 23)	
13 sec.		141.58 (n = 7)	
15 sec.			306.19 (n = 47)
35 sec.	84.99 (n = 4)		

Table S2. Complete list of Cormack-Jolly-Seber models evaluated to estimate total stopover duration of untagged Grey-cheeked Thrush, during two spring migrations in northern Colombia. The model which received the highest support is shown in bold font.

Model of total stopover	No. Params	AICc	Δ AICc	*wi*
**Phi(~Date)p(~1)Gamma(~Date)**	**5**	**836.67**	**0.00**	**0.53**
Phi(~Date)p(~1)Gamma(~Date + mass)	6	838.90	2.23	0.17
Phi(~year + Date)p(~1)Gamma(~Date)	7	840.99	4.32	0.06
Phi(~Date)p(~1)Gamma(~Date + year)	7	841.18	4.51	0.06
Phi(~year)p(~1)Gamma(~Date)	6	841.38	4.71	0.05
Phi(~year + mass + Date)p(~1)Gamma(~Date)	8	843.30	6.63	0.02
Phi(~year + Date)p(~1)Gamma(~Date + mass)	8	843.31	6.64	0.02
Phi(~year + mass)p(~1)Gamma(~Date)	7	843.48	6.81	0.02
Phi(~Date)p(~1)Gamma(~Date + year + mass)	8	843.49	6.82	0.02
Phi(~year)p(~1)Gamma(~Date + mass)	7	843.66	6.99	0.02
Phi(~year + Date)p(~1)Gamma(~Date + year)	9	844.65	7.98	0.01
Phi(~year + mass)p(~1)Gamma(~Date + mass)	8	845.18	8.52	0.01
Phi(~year)p(~1)Gamma(~Date + year)	8	845.24	8.57	0.01
Phi(~year + mass + Date)p(~1)Gamma(~Date + mass)	9	845.63	8.96	0.01
Phi(~year + mass + Date)p(~1)Gamma(~Date + year)	10	847.04	10.37	0.00
Phi(~year + Date)p(~1)Gamma(~Date + year + mass)	10	847.06	10.39	0.00
Phi(~year + mass)p(~1)Gamma(~Date + year)	9	847.37	10.70	0.00
Phi(~year)p(~1)Gamma(~Date + year + mass)	9	847.60	10.93	0.00
Phi(~year + mass)p(~1)Gamma(~Date + year + mass)	10	848.95	12.28	0.00
Phi(~year + mass + Date)p(~1)Gamma(~Date + year + mass)	11	849.42	12.75	0.00

Table S3. List of generalized additive models evaluated to describe change in body mass as a function of days since first capture (days). Covariates included date of first capture (date), year, age and the effect of a being tagged. A model including the effect of date and an interaction between days since first capture and year explained 61% of the variation in the data (shown in bold).

Model	AICc	Δ AICc	*wi*
**Δ mass ~ s(days) + s(date) + days*year**	**409.51**	**0.00**	**0.87**
Δ mass ~ s(days) +s(date)	414.76	5.25	0.06
Δ mass ~ s(days) + s(date) + days*Tagged	415.46	5.95	0.04
Δ mass ~ s(days) + days*year	418.16	8.66	0.01
Δ mass ~ s(days) + s(date) + days*Tagged + days*age	419.21	9.70	0.01
Δ mass ~ s(days) + days*Tagged	420.18	10.67	0.00
Δ mass ~ s(days) + days*age	421.82	12.31	0.00
Δ mass ~ s(days) + days*Tagged + days*age	421.96	12.45	0.00

Table S4. Data for birds inferred to have made direct flights from Colombia to North America. The bird with the fastest speed was also the one which flew furthest (in bold).

**Tag ID**	**Age**	**Departure date and time (dd/mm/yy)**	**Hours elapsed**	**Distance flown (Km)**	**Speed (Km/h)**
40	ASY	05/05/2015 19:51	79.41	3249.00	40.91
213	SY	07/05/2016 19:51	20.61	872.36	42.32
**215**	**ASY**	**08/05/2016 21:02**	**46.26**	**3507.01**	**75.81**
335	ASY	12/05/2016 20:34	72.06	2934.20	40.72
330	ASY	15/05/2016 19:52	37.35	2602.08	69.68
326	SY	15/05/2016 20:02	36.90	2602.07	70.52
311	SY	15/05/2016 20:03	38.75	2601.20	67.12
303	SY	16/05/2016 19:56	41.29	2600.99	62.99
325	SY	16/05/2016 20:05	38.60	2601.20	67.39
307	ASY	17/05/2016 19:47	42.38	2615.00	61.71
316	ASY	17/05/2016 20:13	42.04	2600.53	61.86

Table S5. Linear mixed effects models evaluated to predict the pace of migration of Grey-cheeked Thrush from northern Colombia. The best model (in bold) predicted significant effects of departure fuel load (DFL), Region of the detection, date of departure from Colombia and a random effect of bird ID.

Model of pace of migration	AICc	ΔAICc	*wi*	
**Pace ~ DFL + Region + Depart + DFL:Region + (1|Tag)**	**350.04**	**0.00**	**0.99**	
Pace ~ DFL + Depart + Region + (1|Tag)	359.65	9.61	0.01	
Pace ~ DFL + Region + DFL:Region + (1|Tag)	371.34	21.30	0.00	
Pace ~ DFL + Region + (1|Tag)	379.29	29.25	0.00	
Pace ~ DFL + Depart + (1|Tag)	404.46	54.42	0.00	
Pace ~ Depart + (1|Tag)	407.72	57.67	0.00	
Pace ~ DFL + Region	425.47	75.43	0.00	
Pace ~ DFL + Region + Region:DFL	427.51	77.47	0.00	
Pace ~ DFL	450.84	100.79	0.00	
**Coefficients of the best model**	**Estimate**	**SE**	**t value**	**P**
DFL	-2.40	5.68	-0.42	< 0.001
Gulf Coast	108.71	15.20	7.15	< 0.001
Midwest	0.98	3.72	0.26	
Ontario	11.47	3.11	3.69	< 0.001
Depart	-0.77	0.13	-6.02	< 0.001
DFL:Midwest	8.21	6.98	1.18	
DFL:Ontario	-3.31	5.66	-0.59	

References

1. Redfern, C. P. F. & Clark, J. A. *Ringers' Manual, 4th ed. British Trust for Ornithology, Tetford, United Kingdom.* (British Trust for Ornithology, 2001).
